# Assessment of Aggression and Anger Levels in Athletes: A Study on Gene Polymorphisms in Forensic Science

**DOI:** 10.3390/genes17010011

**Published:** 2025-12-23

**Authors:** Buse Sabiha Bozaslan, Emel Hulya Yukseloglu, Nazli Holumen, Itir Erkan, Faruk Celik, Murat Diramali, Sermin Durak, Sakir Umit Zeybek

**Affiliations:** 1 Department of Forensic Sciences, Faculty of Engineering and Natural Sciences, Uskudar University, 34662 Istanbul, Türkiye; busesabiha.bozaslan@uskudar.edu.tr; 2 Institute of Forensic Sciences and Legal Medicine, Istanbul University—Cerrahpasa, 34303 Istanbul, Türkiye; yuksel@iuc.edu.tr (E.H.Y.); nazli.holumen@ogr.iuc.edu.tr (N.H.); 3 Department of Occupational Health and Safety, Faculty of Health Sciences, Istanbul Yeni Yuzyil University, 34010 Istanbul, Türkiye; 4 Division of Medical Genetics, Department of Internal Medicine, Faculty of Medicine, Alaaddin Keykubat University, 07425 Antalya, Türkiye; faruk.celik@alanya.edu.tr; 5 Division of Anatomy, Department of Basic Medical Sciences, Faculty of Medicine, Bolu Abant Izzet Baysal University, 14030 Bolu, Türkiye; mdiramali@ibu.edu.tr; 6 Department of Medical Microbiology, Faculty of Medicine, Istanbul University—Cerrahpasa, 34320 Istanbul, Türkiye; sermin.dag@iuc.edu.tr; 7 Department of Molecular Medicine, Aziz Sancar Institute of Experimental Medicine, Istanbul University, 34093 Istanbul, Türkiye; uzeybek@istanbul.edu.tr

**Keywords:** behavioral genetics, forensic science, kickbox, aggression, violence

## Abstract

**Background/Objectives**: Many studies in the literature are increasingly focusing on how genes influence the development of individual behaviors and personality traits through genome sequencing. Most research indicates that complex behaviors and their characteristics are influenced by multiple genes, highlighting the crucial role of genetic studies in this field. Behavioral genetics, as a scientific discipline, investigates how genetic factors shape individuals’ behaviors and personality traits. The concepts of violence and aggression, observable in various contexts, have been extensively studied, with a particular focus on the underlying causes of these behaviors. In sports, where physical strength plays a significant role, regulations designed to prevent violent behaviors and aggressive attitudes contribute to the establishment of appropriate behavior patterns and discipline. **Methods**: This study aims to identify correlations between polymorphisms found in athletes and their responses to questionnaires, focusing on candidate genes known to influence personality and behavior traits, such as *catechol-O-methyltransferase (COMT)*, *serotonin transporter (5-HTT)*, *monoamine oxidase (MAO-A)*, and *serotonin 1A transporter (5-HT1A)*. A total of twenty licensed athletes participated in the study. Participants completed three standardized instruments: the Sportsmanship Behavior Scale (27 items), the Sports Emotion Scale (22 items), and the Anger-Control Scale (34 items). Following the acquisition of informed consent, buccal swab samples were collected for single nucleotide polymorphism (SNP) analysis targeting the *COMT, MAO-A, 5- HT1A, and 5-HTT genes*. Subsequent to sample collection and questionnaire administration, statistical analyses were conducted to evaluate the relationships among behavioral measures and genetic variants. **Results**: Overall, the findings point to gene-specific patterns in *5-HTT, MAO-A*, and *COMT*, while no clear pattern emerged for *5-HT1A*. **Conclusions**: Ultimately, this study provides an early exploration of aggression-related genetic patterns within the context of forensic sciences, highlighting preliminary trends and potential associations that may inform the design of future research.

## 1. Introduction

Violent and aggressive behaviors, and the reasons behind them, have been widely studied because they are seen in many parts of life. Human aggression is commonly categorized into proactive and reactive types. Proactive aggression involves reduced emotional sensitivity and is not accompanied by premeditated regret or sadness. On the other hand, reactive aggression is characterized by heightened emotional sensitivity and is triggered by negative emotions such as anger, anxiety, or adverse experiences [1].

In most studies examining aggressive behavior from both neurological and genetic perspectives, it is believed that the interplay between an individual’s genetic makeup and their environment contributes to the variability in aggressive behavior among individuals. Consequently, genetic effects have been found to be more pronounced in those exhibiting deliberate aggression compared to those displaying reactive aggression. This highlights that the most promising results in genetic research on aggression are seen in individuals with deliberate aggression [2].

When examining individuals who engage in violence across different societies, it is clear that there are fundamental differences in the causes and contexts of violence. Studies on sports genetics include identifying genes that influence athletic performance, understanding the mechanisms through which these genes function, and determining genetic predispositions related to athletic abilities. These studies also explore the underlying behaviors observed in sports competitions [3].

In the literature, studies aimed at identifying genetic variants associated with a predisposition to aggression and anger have generally used two approaches: candidate gene association studies (CGAS) and genome-wide association studies (GWAS). Most studies investigating patterns of aggressive behavior and their phenotypes have focused on candidate genes, selected based on previous research or biological plausibility. Many of these genes encode proteins, enzymes, or receptors involved in dopamine or serotonin neurotransmission. In contrast, only a limited number of large-scale, hypothesis-free scans have explored the various phenotypes associated with aggression, facilitating the identification of novel genes and highlighting potential new pathways and functions related to these behaviors [4,5]. The *MAO-A* gene encodes monoamine oxidase A, which is responsible for the catabolism of dopamine, serotonin, and norepinephrine. Located on the X chromosome, this gene has been predominantly studied in males or has shown gene variations in male samples. Interpreting results in female subjects is challenging due to limited information on X chromosome inactivation. The 30-bp variable number tandem repeat (VNTR) in the promoter region has been identified as the most extensively studied genetic variant associated with aggressive behavior. This VNTR, which leads to reduced transcriptional activity, is linked to low-activity alleles and is associated with aggressive behavior and heightened aggression traits, including impulsivity, reactive aggression, violent behavior, criminal activity, weapon use, stabbing, and shooting [6,7].

The *COMT* gene codes for catechol-O-methyltransferase, which is involved in the metabolism of dopamine, epinephrine, and norepinephrine. The most extensively studied variation, Val158Met, with the Val/Val genotype, has been associated with aggression, externalizing behaviors, and anger in several studies. However, other research has not replicated these findings or has indicated that the Met allele is linked to anger and aggressive behavior [8].

One of the most comprehensive meta-analyses of candidate gene studies to date has found no consistent or significant associations with aggression [9]. Genome-wide association studies (GWAS) of aggression have been insufficient in identifying common variants with small effect sizes associated with complex phenotypes. Twin and adoption studies have examined various types of aggressive behaviors, uncovering notable differences between reactive and proactive aggression, as well as direct and indirect aggression. Different types of aggression are believed to reflect distinct underlying mechanisms; for instance, more deliberate, proactive forms of aggression, characterized by goal-directed and purposeful behavior, appear to be more strongly influenced by genetic factors than reactive aggression [10]. Our analysis was based on studies in which aggression was the primary outcome or diagnosis, aiming to provide an exploratory perspective on this topic. Furthermore, this study represents an early investigation of aggression-related genetic patterns within the context of forensic sciences, potentially contributing to future research in this area.

## 2. Materials and Methods

### 2.1. Participant

This study was a prospective design, conducted at the gym licensed by the Turkish Kickboxing Federation. Twenty licensed athletes participated in the study. Kickboxing was selected for this study due to its unique combination of high physical demands and the requirement for substantial mental focus and emotional control. Combat sports, including kickboxing, are characterized by repeated exposure to high-stress situations and rapid decision-making under pressure, which can influence both psychological and physiological responses [11]. Furthermore, the competitive nature of kickboxing necessitates the regulation of aggressive impulses and emotional states, making it a suitable model for examining the interaction between genetic polymorphisms and behavioral traits [12].

The study was explained to the athletes and samples were taken after signing the informed consent form (with approval from the IU-Cerrahpasa ethics committee, numbered E-59491012-604.0102-2636 and dated 5 January 2021). The study included 20 licensed athletes (8 females and 12 males) aged 18–39. Although the sample size was modest, the research was designed as a preliminary exploratory study focusing on four candidate genes. Due to the limited sample size and small effect sizes, the findings should be considered preliminary, providing initial insights for future studies.

### 2.2. Study Tools

Participants, university students engaged in sports activities, completed three instruments assessing emotions, sportmanship behaviors, and aggression in sports contexts. Emotions were measured using the 22-item Sport Emotion Questionnaire (SEQ) [13], which assesses anger, anxiety, dejection, excitement, and happiness on a 5-point Likert scale; the Turkish adaptation demonstrated acceptable factorial validity and internal consistency (α = 0.77–0.87). Sportmanship was evaluated with the 27-item Sportsmanship Behavior Scale [14], covering subdimensions of rules, deliberate behavior, opponent, perspective on the game, and sportsmanship behavior, also using a 5-point Likert format; previous studies support its validity and reliability for university students and sport practitioners. Participants’ levels of anger and their anger expression styles were assessed using two psychometric instruments. The Trait Anger Scale [15] consists of 10 items and evaluates individuals’ general tendencies toward anger. The Anger Expression Style Scale comprises 24 items and includes three subscales: outwardly expressed anger (8 items), inwardly directed anger (8 items), and anger control (8 items). In the Turkish adaptation, Cronbach’s α coefficients were 0.79 for trait anger, 0.84 for anger control, 0.78 for outwardly expressed anger, and 0.62 for inwardly directed anger. These instruments are considered reliable for assessing both the levels and expression styles of anger in this sample. After obtaining consent, an oral swab sample was collected for DNA analysis for specific SNP loci. The sample was then transported directly to the laboratory, maintaining cold chain conditions throughout the process.

Oral swab samples from 20 different individuals were transferred to the laboratory in DNA stabilization tubes, where lysis buffer was added, and the samples were stored at +4 °C. The DNA samples were prepared for isolation procedures after being appropriately transferred with the necessary solutions. Optimization continued until the DNA was fully isolated (DNA isolation kit Mn-net, Macharey-Nagel, Germany), and the DNA quantification was performed by using a spectrophotometer.

The next step involved proceeding to the PCR stage to detect gene polymorphisms in the purified products. The samples were then subjected to the necessary PCR cycles for amplification. For each SNP, one pair of wild-type genomes was amplified. Primers and probe sets were also used to amplify mutated targets for each SNP. Forward and reverse primers (Sentebiolab, Ankara, Turkey) were then designed and employed, and the process continued using these primer sets. Using the designed primers, genomic DNA samples were subjected to PCR by maintaining constant temperatures on thermal cyclers (CFX96 Touch Real-Time PCR Detection System, BioRad, CA, USA). The samples were placed in PCR tubes (vNAT® Transfer Tube (Bioeksen, Istanbul, Turkey) with volumes specified in the table, and optimization was performed according to the established protocol. DNA samples were added to PCR mixtures prepared at appropriate concentrations, and a procedure for the PCR reaction was established under optimal conditions. Four different PCR conditions were applied to address the various polymorphisms of all genes. Following the cycles performed by real-time PCR (SYBR green master mix, PCR 372, Jena Bioscience, Germany), only for the *MAO-A* PCR products were separated based on variant types using agarose gel electrophoresis. To verify the successful amplification of the desired PCR products for the *COMT, MAO-A, 5-HT1A*, and *5-HTT* genes, melting peaks were assessed. RT- PCR protocols were optimized and executed for 45 cycles. It was confirmed that the desired PCR products were obtained in all experiments.

### 2.3. Statistical Analysis

The responses were processed for statistical analysis and included in the statistical procedures to determine correlations. Distribution of our data was evaluated with Shapiro–Wilk test. The data were analyzed using the Kruskal–Wallis test to assess whether there were significant differences between the means of two or more samples. Mann–Whitney U tests were with Bonferroni correction used for comparisons between subgroups, and correlation levels were calculated.

## 3. Results

Gene frequencies were calculated across the entire sample, and median values for the scales were determined based on participants’ responses to the survey questions. Correlation analyses were conducted using results obtained from validated and reliable scales. Data deviating from a normal distribution were identified and considered in the analyses.

It was observed that only a limited number of alleles and parameters yielded statistically significant results. Therefore, the presented data should be considered preliminary, and interpretations of the findings were approached with caution. Particular attention was paid to the limited sample size, and all statistical relationships derived from these early study findings are detailed in the corresponding tables.

### 3.1. Monoamine Oxidase-A Gene (MAO-A)

Table 1 and Table 2 present the statistically significant values and post hoc analyses for genes exhibiting normal and non-normal distributions among polymorphic variants in the population. The frequencies of the *MAO-A* gene variants 3, 4, and 5 were 0.2, 0.45, and 0.35 percent, respectively.

Analyses presented in Table 1 suggest that, for the Anger-Out parameter of the Anger Control Scale, preliminary observations of the *MAO-A* gene may indicate a potential association. For the Continuous Anger parameter, which did not follow a normal distribution, individuals carrying the 5-3 allele similarly demonstrated limited significance. These findings imply a potential relationship between genotype and behavioral tendencies and provide preliminary support for the proposed hypothesis. Additionally, similar studies have reported significant polymorphic effects in variants associated with low expression.

When examining data that did not conform to a normal distribution, *MAO-A* gene alleles were observed to exhibit preliminary effects on the Continuous Anger parameter. Post hoc analyses revealed that, when comparing allele pairs, variants 5 and 3 showed limited significance, with these observations providing preliminary evidence for the study.

### 3.2. Catechol-O-Methyltransferase Gene (COMT)

Table 3 presents the statistically significant values and Post Hoc analyses of genes with normal and non-normal distributions among genes with polymorphisms in the population. For the *COMT* gene, the frequencies of the GG, AA, and AG alleles were 0.3, 0.25, and 0.45 percent, respectively.

Analyses of the *COMT* gene suggest that all alleles (GG, AG, AA) may be associated with preliminary effects on the Anger-Out parameter. For the Anger Control Scale, significance was observed in individuals with GG and AG alleles, and these results followed a normal distribution, indicating limited preliminary evidence that these genotypes could be related to anger regulation.

In contrast, for the Persistent Anger scale, which did not follow a normal distribution, significance was observed in individuals carrying the AA and GG alleles, and these findings can be interpreted as preliminary. These results are generally consistent with the hypothesis and align with trends reported in similar studies in the literature. When examining data that did not conform to a normal distribution, *COMT* gene alleles also showed significant values for the Continuous Anger parameter. Post hoc analyses (Table 4) revealed that, in pairwise comparisons of the alleles, the AA and GG variants were significantly observed, and these observations provide limited preliminary evidence of potential genotype–behavior associations.

**Table 4:** Post hoc analysis of data that do not follow a normal distribution based on the COM-T gene.

Persistent Anger					
Sample 1-Sample 2	Test Statistic	Std. Error	Std. Test Statistic	Sig.	Adj. Sig.
**AA-AG**	6.300	3.534	1.783	0.075	0.224
**AA-GG**	12.057	3.777	3.192	0.001	**0.004**
**AG-GG**	5.757	3.179	1.811	0.070	0.210

>0.05: Nonsignificant; <0.05: Significant; <0.01: Highly significant.

### 3.3. 5-Hydroxytryptamine Transporter Gene (5-HTT)

Table 5 included a comparison of data that do not follow a normal distribution based on the *5-HTT* gene in this population, and it presents the statistically significant values and Post Hoc analyses of genes with normal and non-normal distributions among genes with polymorphisms in the population. For the *5-HTT* gene, the frequencies of the GG, AA, and AG alleles were 0.25, 0.4, and 0.35 percent, respectively.

Analysis of the *5-HTT* gene showed significant values for the GG and AA alleles in the Anger-Out parameter, suggesting preliminary associations. Unlike other genes, significant scores were obtained for all alleles (GG, AG, AA) in the Happiness parameter, indicating a potential relationship between these genotypes and affective expression. However, no data that did not follow a normal distribution was detected; as a result, this aspect was not considered in the statistical analyses due to the nature of the calculations, and the findings should be interpreted as providing limited preliminary evidence.

### 3.4. 5-Hydroxytryptamine Receptor -1A Gene (5-HT1A)

Table 6 and Table 7 present the statistically significant values and Post Hoc analyses of genes with normal and non-normal distributions among genes with polymorphisms in the population. For the *5-HT1A* gene, the frequencies of the GG, CC, and CG alleles were 0.25, 0.4, and 0.35 percent, respectively.

Upon examining the *5-HT1A* gene, no significant results were found in any of the other data, except for the C allele, where a preliminary association was observed with the Persistent-Anger parameter. Since no significant findings were detected in other variants or in any pairwise evaluations, these were not included in the parallel analysis. Although only certain parameters yielded significant results, these findings, which are consistent with our hypothesis, may provide preliminary insights and help guide future research, potentially contributing to a new dataset in the literature.

## 4. Discussion

Numerous studies have suggested that dopaminergic functions may play a role in shaping individual behavior. In athletes, personal performance is influenced by genetic factors and likely reflects the interaction between inherent potential and training. Sports performance is generally considered to result from the interplay between genetic predisposition and structured training. Athletic success is facilitated by the integration of talent identification and training management systems, which are thought to contribute to the development of athletic potential [16]. Parameters such as anger and aggression are recognized as influential factors in athletic performance, affecting both athletes’ daily functioning and their competitive outcomes. Given that anger can substantially impact performance, effective anger regulation is considered essential for achieving successful results in sports [17].

In this context, identifying characteristics such as stress, competition, anxiety, anger, and aggression—which have been suggested to influence sports performance and are thought to be associated with serotonergic, dopaminergic, and non-androgenic systems—may be meaningful for understanding athletic behavior. Accordingly, examining genetic factors that are considered to contribute to these psychological states may provide preliminary insights into individual differences in athletic performance [18]. In addition, epigenetic changes arising from environmental influences and lifestyle-related factors may modulate gene expression, potentially playing a role in shaping individual behavioral patterns and phenotypic traits over time. Furthermore, environmental conditions may influence the expression of specific polymorphisms or be associated with the emergence of more adaptive behavioral responses [19]. In this study, gene frequencies were calculated in a sample of 20 licensed kickboxing athletes to explore the prevalence of different genetic variants. To investigate potential correlations with behavioral patterns, data were collected using the Anger-Control, Sportsmanship Behavior, and Sports Emotions scales. Considering that some results did not conform to a normal distribution and the relatively small sample size, this study was conducted as an exploratory pilot investigation, applying multiple statistical tests to identify preliminary observations. Additionally, the similarities in athletes’ training programs and disciplinary routines suggest that the influence of genetic factors on behavioral traits is likely shaped by multiple parameters rather than a single polymorphism. Accordingly, the findings are interpreted as preliminary observations, and further studies with larger samples and diverse sports populations are recommended to validate and extend these results.

In behavioral genetics research, catecholamines like dopamine and norepinephrine, produced in the human body, are involved in neurotransmission within both the central and peripheral nervous systems. These chemicals are mainly metabolized by monoamine oxidase (MAO), and this pathway has been shown to play a significant role in shaping personality traits [20].

In regulating the dopaminergic system, impulsive aggressive behaviors are known to be linked to disruptions in the balance of neurotransmitters in the prefrontal cortex, particularly within the serotonergic system. Furthermore, serotonin, which has various effects on both the central and peripheral nervous systems, is an endogenous molecule involved in regulating behavior. This neurotransmitter has been associated with behaviors such as obsession, depression, addiction, and anxiety [21].

Our study suggests that *MAOA* and *COMT* gene variants may contribute modestly to individual differences in aggression and emotional regulation among athletes, consistent with previous literature. Monoamine oxidase A (MAOA) and catechol-O-methyltransferase (COMT) are key enzymes in catecholamine metabolism, regulating dopamine, norepinephrine, and serotonin [22,23]. The *MAOA* gene, located on the X chromosome, contains a VNTR polymorphism in its promoter that modulates transcriptional activity. Alleles with 3.5 and 4 repeats are generally associated with higher expression, whereas 2 and 3 repeats are linked to lower expression [22,23,24,25,26,27]. The functional impact of the 5-repeat allele remains less clear and may vary across populations.

The *COMT* gene polymorphism influences enzymatic activity and dopamine metabolism, which can affect behavioral traits [24]. Previous studies indicate that gene-environment interactions may play a more critical role than individual polymorphisms in determining behavioral outcomes such as aggression or impulsivity [24,25,26]. While low-activity *MAOA* alleles have been associated with higher susceptibility to aggressive behaviors under certain environmental conditions, these effects are modest and not consistently replicated across populations [24,26].

In our study, eight athletes were identified as carriers of the high-expression 4-repeat and wild-type *MAOA* alleles, while the remaining twelve athletes carried low-expression alleles associated with the 3- and 5-repeat variants. Associations between *MAOA* 3-, 4-, and 5-repeat alleles and the subscales of the Anger-Control Scale (Persistent Anger, Anger-In, Anger-Out, Anger-Control) as well as the Sport Emotion Scale subscales are summarized in Table 1. Post hoc pairwise comparisons for Persistent Anger revealed that the 5-3 variant was significantly associated with the outcomes. These findings are consistent with previous literature reporting associations between low-expression alleles and anger-related behavioral tendencies, providing an early indication that warrants further investigation in this area (Table 2).

For the Anger-Out subscale, which exhibited a normal distribution, individuals with the 3- and 4-repeat alleles showed similar preliminary results. This suggests a potential relationship between *MAOA* genotype and behavioral traits related to outward-directed aggression. In our sample, the wild-type and low-expression 3-repeat alleles were more frequently observed. Considering the limited sample size, these findings provide preliminary evidence aligned with previous studies and offer guidance for future research with larger cohorts.

Furthermore, Shapiro–Wilk tests for non-normally distributed Persistent Anger scores showed significant associations for individuals carrying the 5-3 alleles, indicating a preliminary exploratory relationship between genotype and anger-related behavior, despite the small sample size. Overall, these results suggest that *MAOA* genotypes may play a potential role in the regulation of anger and aggression, providing early and exploratory evidence in this context.

Research indicates that the *COMT* gene is linked to an increased risk of behavioral disorders and mental illnesses, as structural changes in the prefrontal cortex are associated with this gene. Genetic research not only examines these genes but also includes studies on many other genes associated with hereditary traits related to anxiety, aggression, and depression. Previous studies have linked the *COMT* gene polymorphism to psychological and behavioral traits in athletes. In a study with a small sample of 16 combat sport athletes and 40 control participants, the relationships between gene polymorphisms, stress resilience, personality traits, and aggression tendencies were examined. Some athletes were observed to carry the Val/Val (GG) genotype at a higher frequency compared with the control group [28].

In another study with a larger sample, including 258 combat sport athletes and 278 controls, analyses similarly revealed significant associations between the *COMT* rs4680 genotypes and behavioral trait dimensions (novelty seeking, self-management, and self-transcendence). These findings suggested that *COMT* variants may influence psychological profiles and could be differently represented among athletes [29].

Moreover, another study reported that the Met (A) allele of the *COMT* rs4680 polymorphism reduces enzymatic activity, which is associated with increased prefrontal dopamine levels in healthy individuals, consequently affecting performance on motor control tasks. This mechanistic insight highlights the potential for *COMT* polymorphisms to modulate dopaminergic signaling, thereby potentially influencing cognitive and behavioral traits in athletes [30]. In our sample, only five athletes carried the wild-type allele, whereas the remaining fifteen individuals carried either the mutant or heterozygous forms. Despite the limited sample size, this distribution is consistent with previous reports indicating a higher prevalence of the mutant allele compared to the wild-type allele in similar populations.

Analyses of the *COMT* gene revealed that all alleles (GG, AG, AA) were associated with the Anger-Externalizing scale. Regarding the Anger-Control scale, individuals carrying GG and AG alleles exhibited more pronounced associations in normally distributed data compared to other participants, suggesting preliminary evidence that these genotypes may be linked to a greater capacity for anger regulation relative to AA carriers. For non-normally distributed measures, significant associations were observed among homozygous AA and GG individuals on the State-Labile Anger scale, indicating early trends toward a relationship between genotype and the tendency to experience persistent anger (Table 3).

Pairwise comparisons further showed that the G allele was significantly associated with Anger-Externalizing and Anger-Control parameters, whereas the A allele was significantly associated with Anger-Externalizing. These findings, consistent with normally distributed measures, suggest that homozygous carriers may exhibit higher scores on externalized anger behaviors, reflecting preliminary genotype-behavior associations (Table 4).

Serotonin (5-HT) is thought to act as a hormone that promotes feelings of happiness, vitality, and well-being, and it plays a crucial role in regulating anxiety. A deficiency in serotonin can result in moods characterized by depression, fatigue, and irritability. When serotonin is released in the brain, it causes blood vessels to constrict, while a decrease in serotonin levels leads to vasodilation [31].

The serotonin transporter protein SERT, also known as 5-hydroxytryptamine transporter (5-HTT), is encoded by the *SLC6A4* gene and is responsible for the reuptake of serotonin from the synaptic cleft. A functional polymorphism in the promoter region, called 5-HTTLPR, has been linked to aggressive behavior. Specifically, the short allele (S) and SS genotypes, which lead to lower transcription levels, have been associated with increased aggression, neuroticism, hostility, anger, impulsive aggression, enmity, violent behavior, and criminality. Research suggests that the S allele may be related to anxiety, and the SS genotype could be associated with aggression in children [32]. In our study, significant associations were observed between the GG and AA alleles and the Anger-Externalized parameter, as well as significant effects on the Happiness parameter. These findings are consistent with previous literature, indicating that individuals carrying these polymorphisms tend to express both anger and happiness more prominently. Furthermore, given that prior studies have reported fewer direct associations with happiness, these results may represent a novel contribution. However, confirmation of these preliminary findings will require replication in a larger sample.

However, the relationship between behavior and the polymorphisms of genes involved in the 5-HT signaling pathway, such as the *5-HTT*, the *HTR1A*, and *MAO-A*, has been the primary focus of investigation. In the literature, the wild-type allele has generally been reported to be more prevalent in healthy individuals and associated with positive mood rather than stress. In our study, the wild-type allele was observed in eight individuals and was associated with the Happiness parameter. This finding, consistent with some previous reports, indicates that although the wild-type allele was observed in a small number of participants, higher Happiness scores were recorded in Table 5 suggesting a preliminary association between the wild-type allele and a more positive affective state [33,34,35,36].

The *5-HT1A* gene has been highlighted in the literature for its relationship with serotonin receptors and its potential involvement in conditions such as anxiety and depression [37]. Table 6 indicates that, while this result may be consistent with some previous findings, further studies with larger sample sizes are needed. In our study, a significant association was found only with the C allele for the Persistent Anger parameters (Table 7). These data should be considered exploratory, providing preliminary insights and guiding future research directions.

## 5. Conclusions

In this study, gene frequencies were calculated in a sample of 20 licensed kickboxing athletes to investigate the prevalence of different genetic variants. Data were collected using the Anger-Control, Sportsmanship Behavior, and Sports Emotions scales to examine potential associations with behavioral patterns. Considering that some results did not conform to a normal distribution and the relatively small sample size, this study was conducted as an exploratory pilot investigation, applying multiple statistical tests to identify preliminary findings. Moreover, the similarities in athletes’ training programs and disciplinary routines suggest that the influence of genetic factors on behavioral traits is likely shaped by multiple parameters rather than a single polymorphism. Accordingly, the findings are interpreted as preliminary observations, and further studies with larger samples and diverse sports populations are recommended to validate and extend these results.

Despite these limitations, this study, derived from a doctoral thesis, aims to provide insight for more comprehensive research with larger sample sizes, as well as to emphasize the importance of genetic studies in the field of forensic sciences. To address this, our study focused on collecting data of a preliminary nature to elucidate the complex structural sources of anger- and aggression-related behaviors. In conclusion, given the limited number of individuals and specific SNP regions analyzed in this study, it is anticipated that future research with larger sample sizes and broader genomic coverage will make significant contributions to the field.

Violence, which is frequently examined within the field of forensic sciences, was investigated by additionally considering behavioral traits such as aggression and anger attitudes, with the aim of exploring how these characteristics are distributed at the genotypic level among licensed athletes. The findings obtained within this framework should be regarded as preliminary, given that the study was conducted on a limited sample, and are intended to provide a basis for future research involving larger and more diverse populations.

Furthermore, candidate genes identified in the existing literature were included in the present study and examined as biological indicators. In light of the findings, it is emphasized that genetic factors, within a forensic context, cannot be considered criminological markers that independently eliminate criminal responsibility or solely explain criminal behavior within the criminal justice system. Rather, behaviors relevant to the criminal justice process are shaped by a complex interplay of biological, psychological, and environmental factors, as consistently highlighted in the literature [38].

## Figures and Tables

**Table 1:** Comparison of data that do not follow a normal distribution based on the MAO-A gene.

	3				4				5					
	Min	Max	Median	IQR	Min	Max	Median	IQR	Min	Max	Median	IQR	χ^2^	Sig.
**Persistent Anger**	21.00	28.00	23.50	5.00	18.00	27.00	21.00	4.00	6.00	25.00	18.00	14.00	**8.464**	**0.015**
**Tension**	0.20	2.40	1.30	1.75	0.80	2.80	2.00	1.70	0.80	2.60	1.00	1.60	2.291	0.318
**Unpleasantness**	0.00	1.60	0.80	1.00	0.00	2.80	0.80	2.20	0.20	1.60	0.60	0.80	0.498	0.779

>0.05: Nonsignificant; <0.05: Significant; <0.01: Highly significant.

**Table 2:** Post hoc analysis of data that do not follow a normal distribution based on the MAO-A gene.

Persistent Anger					
Sample 1-Sample 2	Test Statistic	Std. Error	Std. Test Statistic	Sig.	Adj. Sig.
**5-4**	3.746	3.251	1.152	0.249	0.748
**5-3**	10.357	3.589	2.886	0.004	**0.012**
**4-3**	6.611	3.400	1.944	0.052	0.156

>0.05: Nonsignificant; <0.05: Significant; <0.01: Highly significant.

**Table 3:** Comparison of data that do not follow a normal distribution based on the COM-T gene.

	GG				AG				AA					
	Min	Max	Median	IQR	Min	Max	Median	IQR	Min	Max	Median	IQR	χ^2^	Sig.
**Persistent Anger**	20.00	28.00	24.00	6.00	6.00	25.00	21.00	4.00	7.00	19.00	18.00	8.00	**10.258**	**0.006**
**Tension**	0.20	2.80	1.80	2.00	0.60	2.60	1.00	1.15	0.80	2.60	2.20	1.20	1.070	0.586
**Unpleasantness**	0.00	2.60	0.80	1.80	0.00	2.80	0.80	1.05	0.20	1.60	0.40	0.90	1.322	0.516

>0.05: Nonsignificant; <0.05: Significant; <0.01: Highly significant.

**Table 5:** Post hoc analysis of data conforming to a normal distribution based on 5-HTT gene.

Dependent Variable			Mean Difference (I-J)	Std. Error	Sig.	95% Confidence Interval	
						Lower Bound	Upper Bound
Anger-Externalized	GG	AG	−1.025	2.137	1.000	−6.64	4.59
		AA	−5.733	2.091	**0.039**	−11.22	−0.24
	AG	GG	1.025	2.137	1.000	−4.59	6.64
		AA	−4.708	1.822	0.055	−9.49	0.07
	AA	GG	5.733	2.091	**0.039**	0.24	11.22
		AG	4.708	1.822	0.055	−0.07	9.49
Happiness	GG	AG	−0.77500	0.27889	**0.036**	−1.5071	−0.0429
		AA	−0.95556	0.27287	**0.007**	−1.6719	−0.2392
	AG	GG	0.77500	0.27889	**0.036**	0.0429	1.5071
		AA	−0.18056	0.23771	1.000	−0.8046	0.4435
	AA	GG	0.95556	0.27287	**0.007**	0.2392	1.6719
		AG	0.18056	0.23771	1.000	−0.4435	0.8046

>0.05: Nonsignificant; <0.05: Significant; <0.01: Highly significant.

**Table 6:** Comparison of data that do not follow a normal distribution based on the 5-HT1A gene.

	CC				CG				GG					
	Min	Max	Median	IQR	Min	Max	Median	IQR	Min	Max	Median	IQR	χ^2^	Sig.
**Persistent Anger**	7.00	22.00	18.50	5.00	6.00	28.00	23.00	7.00	18.00	25.00	21.00	6.00	5.198	0.074
**Tension**	0.60	2.60	1.60	1.70	0.20	2.80	1.80	1.60	0.80	2.40	1.00	1.40	0.728	0.695
**Unpleasantness**	0.00	2.80	0.50	1.20	0.00	2.60	0.80	1.80	0.40	1.20	0.80	0.50	0.536	0.765

>0.05: Nonsignificant; <0.05: Significant; <0.01: Highly significant.

**Table 7:** Comparison of normally distributed data according to the C allele of the 5-HT1A gene.

	CC				CG + GG				Z	Sig.
	Min	Max	Median	IQR	Min	Max	Median	IQR		
Persistent Anger	7.00	22.00	18.50	5.00	6.00	28.00	22.50	6.00	**86.500**	**0.035**
Tension	0.60	2.60	1.60	1.70	0.20	2.80	1.40	1.45	59.000	14.548
Unpleasant-ness	0.00	2.80	0.50	1.20	0.00	2.60	0.80	0.95	66.000	0.525

>0.05: Nonsignificant; <0.05: Significant; <0.01: Highly significant.

## Data Availability

The original contributions presented in the study are included in the article, further inquiries can be directed to the corresponding author.
